# Investigation of the seasonal microbiome of *Anopheles coluzzii* mosquitoes in Mali

**DOI:** 10.1371/journal.pone.0194899

**Published:** 2018-03-29

**Authors:** Benjamin J. Krajacich, Diana L. Huestis, Adama Dao, Alpha S. Yaro, Moussa Diallo, Asha Krishna, Jiannong Xu, Tovi Lehmann

**Affiliations:** 1 Laboratory of Malaria and Vector Research, NIAID, NIH, Rockville, Maryland, United States of America; 2 Malaria Research and Training Center (MRTC), Faculty of Medicine, Pharmacy and Odonto-stomatology, University of Sciences, Techniques and Technologies, Bamako, Mali; 3 Biology Department, New Mexico State University, Las Cruces, New Mexico, United states of America; Swedish University of Agricultural Sciences, SWEDEN

## Abstract

The poorly understood mechanisms of dry season persistence of *Anopheles* spp. mosquitoes through the dry season in Africa remain a critical gap in our knowledge of *Plasmodium* disease transmission. While it is thought that adult mosquitoes remain in a dormant state throughout this seven-month dry season, the nature of this state remains unknown and has largely not been recapitulated in laboratory settings. To elucidate possible connections of this state with microbial composition, the whole body microbiomes of adult mosquitoes in the dry and wet seasons in two locations of Mali with varying water availability were compared by sequencing the 16S ribosomal RNA gene. These locations were a village near the Niger River with year-round water sources (N’Gabakoro, “riparian”), and a typical Sahelian area with highly seasonal breeding sites (Thierola Area, “Sahelian”). The 16S bacterial data consisted of 2057 sequence variants in 426 genera across 184 families. From these data, we found several compositional differences that were seasonally and spatially linked. Counter to our initial hypothesis, there were more pronounced seasonal differences in the bacterial microbiome of riparian, rather than Sahelian areas. These seasonal shifts were primarily in *Ralstonia*, *Sphingorhabdus*, and *Duganella* spp. bacteria that are usually soil and water-associated, indicating these changes may be from bacteria acquired in the larval environment, rather than adulthood. In Sahelian dry season mosquitoes, there was a unique intracellular bacteria, *Anaplasma*, which likely was acquired through non-human blood feeding. Cytochrome B analysis of blood meals showed greater heterogeneity in host choice of *An*. *coluzzii* independent of season in the Thierola area compared to N’Gabakoro (77.5% vs. 94.6% human-origin blood meal, respectively), indicating a relaxation of anthropophily. Overall, this exploratory study provides valuable indications of spatial and seasonal differences in bacterial composition which help refine this difficult to study state.

## Introduction

While the overall numbers of *Plasmodium-*caused malarial disease have been decreasing since the early 2000s, the spatial extents of transmission have been mostly consistent, including in areas that have highly seasonal disease transmission such as Mali [1]. A major factor towards this is likely the resilience of the *Anopheles* spp. vectors, and their ability to recolonize an area after a 7–8 month dry season [2]. Critical to this recolonization is a long-lived, reproductively depressed life stage of these mosquitoes called aestivation [2,3]. While this phenotype was described in *Anopheles* spp. over 60 years ago, much still remains poorly understood about this state [4]. Previous entomological survey work has shown that the members of the *Anopheles gambiae* s.l. complex have differing strategies for dry season survival [5]. It is likely *An*. *gambiae* s.s. (previously named *An*. *gambiae* S-form) favors migration to areas of permanent water, while *An*. *coluzzii* (previously named *An*. *gambiae* M-form) remains locally as an adult in a persistant state (aestivation) throughout the dry season [5,6]. The adult dormancy phenotype of *An*. *coluzzii*, characterized by a ~7 fold life span extension has been difficult to induce in laboratory mosquito populations [7,8], and efforts in the field to find aestivating mosquitoes while in their refugia have been largely unsuccessful [9]. These difficulties have prevented the study of the mechanistic, climatic, or behavioral underpinnings of aestivation in these mosquitoes. Thus, additional descriptive studies of *An*. *coluzzii* while in this state, and how they differ from mosquitoes in other seasons or locations with year-round breeding potential (permanent water), but similar climactic conditions would be beneficial to understanding the behaviors of these vectors.

Work on the microbiome of insects has shown that a variety of life history traits are reflected within this complex bacterial ecosystem. Specifically, the microbiome contains signatures which may link mosquito populations geographically [10,11]; it changes in response to bloodfeeding [12], infection with *Plasmodium* [13], and habitat [14]; and shifts inbacterial community compositions have been shown to increase host mortality in *Drosophila* [15]. Recent work has shown there is minor seasonal variation in *An*. *gambiae* s.s. mosquito microbiota from the forest-savannah regions with perennial larval sites during the dry season in Ghana, but that limited differences were found within *An*. *coluzzii* between seasons and locations [16]. However, there has been no work to date that has characterized the variability present in the whole-body mosquito microbiome in areas with more distinct seasonality such as the Sahel where no larval habitat could be found during the dry season.

To investigate seasonal compositional differences in the microbiome that may link to aestivation, this exploratory study utilizes quantitative and qualitative measures to evaluate how the microbiome differs in an area in West Africa with highly seasonal water availability and mosquito abundance. We compare seasonal changes within and between locations, initially hypothesizing that we would find the greatest difference in mosquito microbiota found in Sahelian dry season due to this location having the clearest demarcation between physiological states of reproductive vs. aestivating mosquitoes in the wet and dry seasons, respectively. Additionally, we compared how laboratory mosquitoes compare to these field-caught specimens in their microbial composition.

## Materials and methods

### Mosquito collection and field sites

**Fig 1:**
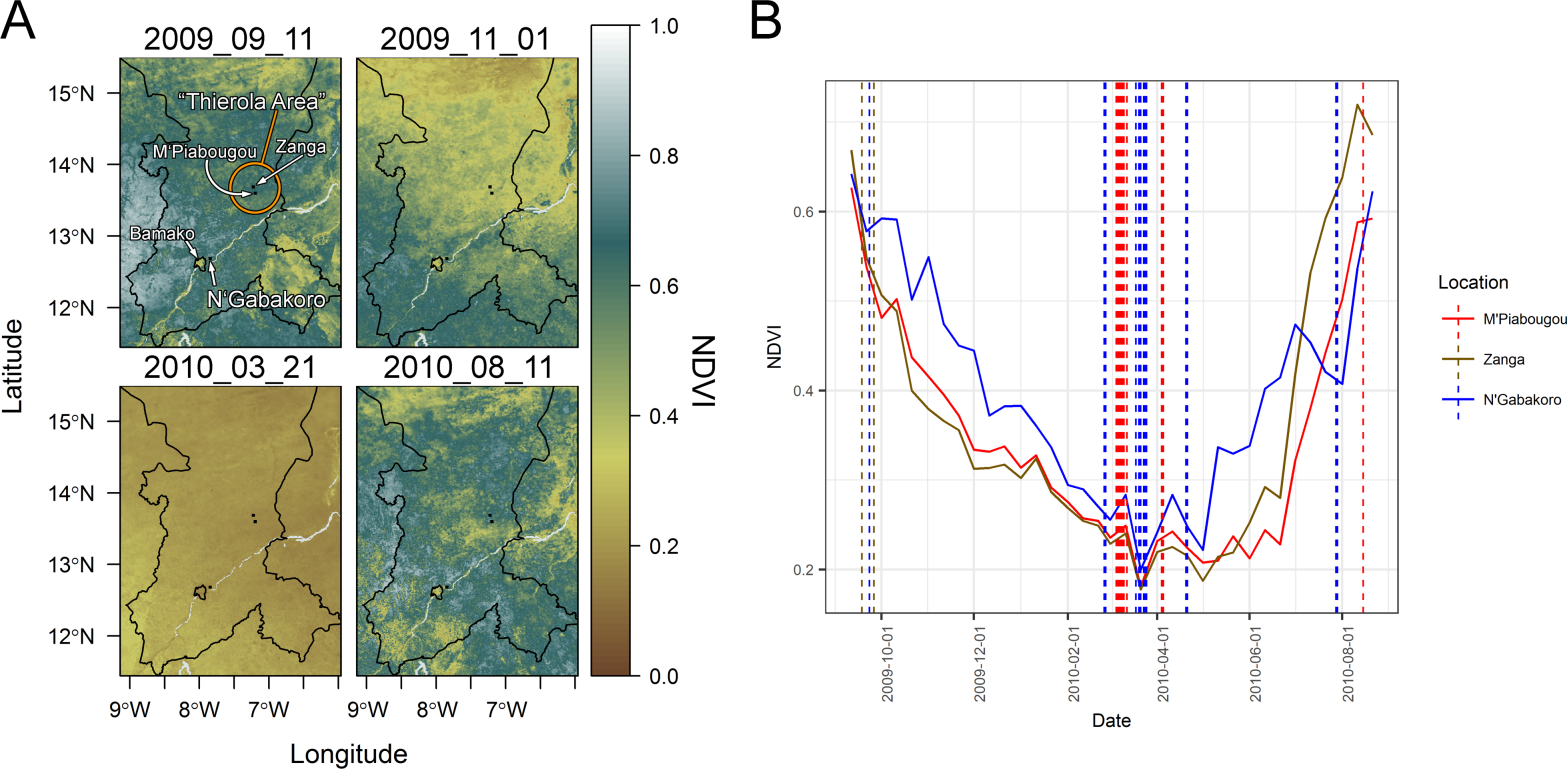
Sampling locations in the Koulikoro region of Mali overlaid over seasonal normalized difference vegetation index data (panel A) and at each location throughout the sampling period (panel B). NDVI measures the greenness (vegetation) level of an area measured from a satellite. The villages of M'Piabougou and Zanga in the area of Thierola with higher seasonality and lower water availability are compared to N'Gabakoro, a village near Bamako and the Niger River using this metric. Examples of wet season (top left, bottom right of panel A), transitional (top right of panel A), and dry season (bottom left panel A) are presented. Microbial sampling dates per village are marked with vertical dashed lines (panel B).

*An*. *coluzzii* mosquitoes were collected via indoor aspiration in three locations, Zanga (Latitude 13.688050°, Longitude -7.221029°), M’Piabougou (13.599830°, -7.192859°), and N’Gabakoro (12.683870°, -7.840419°) in the Koulikoro region of western Mali from September 2009 to August 2010 (Fig 1). Due to the close proximity (~10 km) and similar climates between locations, mosquitoes from Zanga and M’Piabougou were treated as being both in the Sahelian class for analysis. Mosquitoes from each location were noted for blood fed status, had their thoraces punctured to allow for permeation of preservative into the sample, and were added to 50 μl RNA*later* stabilization solution (ThermoFisher Scientific, Waltham, MA, USA). Fourteen additional laboratory reared *An*. *coluzzii* were also preserved in groups of young (3 days old, Samples 55–57) and old (14 days old, Samples 58–60) post emergence to compare microbiome development over time.

Mosquitoes were speciated to molecular form based on a direct PCR performed on two legs with a standard protocol [17]. All mosquitoes were kept in RNA*later* at -80°C until DNA extraction, and all were processed at the same time to limit batch effects. DNA was extracted from mosquitoes with a DNeasy kit according to manufacturer instructions (Qiagen, Valencia, CA, USA), and eluted in 50 μl of the provided elution buffer.

### Normalized difference vegetation index (NDVI) analysis

To further quantify the differences in seasonality between our Sahelian and riparian areas, we calculated the NDVI around the center point of each of the 3 field sampling locations with a ~5 km square on the NDVI output from the 1 km resolution Metop-AVHRR S10 global satellite database (Accessed 5/1/2017, from: http://www.vito-eodata.be/PDF/portal/Application.html#Home) [18]. NDVI contrasts the absorption and reflectance of light to quantify the greenness of plants (live plants reflect near-infrared radiation to prevent overheating). The images spanning the sampling collection period were analyzed using the ‘raster’, ‘sp’, and ‘rasterVis’ packages in R, and four representative time points are shown (Fig 1A). The values and sampling dates were also plotted using the ‘ggplot2’, and ‘scales’ packages (Fig 1B) [19–23]. Shape files for Mali and the sampling regions were acquired from the Database of Global Administrative Areas (GADM) database with ‘raster’.

### Sequencing and data processing

Next-generation sequencing of the V1-V3 regions of 16S ribosomal RNA gene was performed by MR DNA (Shallowater, TX, USA) on a Roche 454 sequencer using the forward primer “27FMod”- 5’-AGRGTTTGATCMTGGCTCAG-3’ and the reverse primer “519Rmodbio”– 5’-GWATTACCGCGGCKGCTG -3’. Amplification and sequencing conditions used are described in full elsewhere [24]. Mosquitoes were sequenced either as individuals from 15ng of DNA, or from a pool of three mosquitoes (5ng from each) with concentrations being determined by NanoDrop (ThermoFisher).

The .fasta and .qual files with primers and barcodes removed were split and demultiplexed in QIIME v1.9.0 [25]. Fastq files were imported into R version 3.4.0 using the RStudio IDE version 1.0.44 using the ‘dada2’ package [26–28]. Processing using this pipeline largely followed the Bioconductor workflow from Callahan *et al*. [29]. Briefly, data were trimmed and filtered based on quality score, and sequencing error rates were learned on a random sample of *n =* 25 with suggested parameters for 454 data of HOMOPOLYMER_GAP_PENALTY = -1 and BAND_SIZE = 32. Chimeras were removed, and taxa was assigned with a naïve Bayesian classifier and the ‘silva_nr_v123_train_set’ for bacterial 16S rRNA or the UNITE database for fungal ITS formatted for use in dada2 (Silva training set: https://zenodo.org/record/158958, UNITE database (General FASTA Release): https://unite.ut.ee/repository.php) [30–32]. For 16S this was based on a 325nt length amplicon, and for ITS was based on a variable length amplicon with a minimum of 50 nt length.

The sequences were aligned with the ‘DECIPHER’ package in R, output with the ‘phangorn’ package version 2.1.1 into FastTree 2 software for generation of a generalized time-reversible (GTR) maximum-likelihood phylogenetic tree with rescaling of branch lengths and computation of Gamma20-based likelihood [33–35]. This tree was read into R using the ‘ape’ package [36], and then the data were combined with the ‘phyloseq’ package for data manipulation and visualization [37]. After contaminant reads from eukaryotic sources were removed the Shannon index of richness was calculated for each mosquito.

### Differential abundance testing and clustering analysis

Seasonal differences in microbial abundance were analyzed by hierarchical multiple testing with the ‘structssi’ package in R [38]. This is a procedure in which you incorporate the innate structure in the data (in this case the phylogenetic hierarchy of the microbes) to adjust the false discovery rate and improve power in comparing count data. We defer the majority of details of this approach to the parent literature [39], and to its implementation literature for microbiome data [29,38]. Briefly, the hypotheses are organized based on the phylogenetic tree of the data, and tested for differential abundance if the parent hypothesis is found to be significant between groups at a coarse level of discrimination (a ‘hFDR’ rate of 0.75) [29]. This analysis was performed on 16S sequence variant count data that was variance stabilizing transformed by ‘DESeq2’ and shifted so all values are positive. Genera with greater than 1.5 shrunken-log_2_fold change that are found to be significant at an adjusted *p-*value of less than 0.05 are presented.

### Presence/absence analysis for seasonally indicative genera

In addition to analysis of the variance in bacterial abundance described above, we performed two analyses based on the presence/absence of bacterial taxa in mosquitoes, regardless of the abundance. In the first, we performed random forest supervised classification analysis on genus-level data using the ‘caret’ package in R [40–42]. The proximity metric calculated during the random forest model generation was utilized to generate a multi-dimensional scaling plot (MDS) to evaluate the degree of difference and clustering between and within sample groups. Group centroids were compared by pairwise permutation multivariate analysis of variance test (PERMANOVA) with 9999 permutations with Benjamini-Hochberg false discovery rate correction using the ‘RVAideMemoire’ package [43]. Prevalence for each of the sample groups for the top 10 most important genera in the random forest classification model are presented.

Additionally, for field samples we enumerated taxa unique to the Sahelian dry season sample, found their relative frequency (in terms of mosquito hosts), and evaluated how unique taxa are to that sample group (“private”—i.e. found only in that sample group) in SAS 9.4 (SAS Institute Inc., Cary, NC, USA). Using “informative” taxa (excluding rare and ubiquitous taxa), we sequentially tested if the frequency (prevalence) of any informative taxa differed between the dry and wet seasons in the Sahel, followed by testing that the Sahelian dry season differs from riparian dry season, and that it also differed from the riparian wet season in a consistent direction. These tests employed the sequential Fisher’s exact tests (with the last two being one-way tests), only passing genera significant at *p <* 0.1 forwards to the next test (Summarized in S1 Fig). The final results only report those genera whose prevalence difference is in the same direction (i.e. higher in Sahel Dry compared to all other conditions).

### Cytochrome B bloodmeal analysis

The mammalian host source of each mosquito and pool per location was analyzed via a size-discriminate multiplexed cytochrome B PCR [44]. This PCR allows for the identification of bloodmeals from pigs, humans, goat, dog and cows.

## Results

Unlike the Sahelian villages, the proximity of N’Gabakoro to the Niger River allows mosquito breeding year-round. To further assess the seasonal differential in aridity between the Sahelian and riparian villages, we analyzed the normalized difference vegetation index (NDVI, Fig 1). Due to its proximity to the Niger River, we found that there is a desiccation lag-period in which the vegetation index does not drop at the same rate in N’Gabakoro as it does in the two villages that have only rainfall as a water source (Fig 1A top right, Fig 1B). Additionally, while the vegetation minima are reached roughly at the same time in the dry season between sites (late March to early April), the overall NDVI remains higher in N’Gabakoro during the transition periods to and from this dry season low point.

### Sequencing

Of the 58 samples sent for sequencing, 53 returned sequence for 16S and 26 returned sequence for ITS. For 16S sequencing 2643 sequence variants (SVs) were found, though some of these variants aligned to eukaryotic reads in the SILVA database and were filtered, leaving 2057 SVs with 426 genera across 184 families. Many of these 16S reads when searched against the ‘nr’ database using BLAST had hits to *An*. *gambiae* strain PEST sequence [45]. This could indicate some cross-reactivity of the primers with other ribosomal sequence, or mis-annotated reference sequence. The fungal internal transcribed spacer (ITS) region sequencing had limited success in amplification in terms of both average read counts per successful sample (755 vs. 4554 for ITS and 16S, respectively), and in the amount of coverage across locations/seasons. Fungal ITS failed to amplify from any samples in N’Gabakoro or the laboratory samples. Due to this, no comparisons were made between season and location of the fungal microbiome. All ITS reads that were not assigned to genus level (63332/79195 total) were filtered. Of the successful samples, the most abundant fungal genera were *Aspergillus* (20.7% of total reads without *Anopheles*) with presence in 12/21 samples with fungal ITS reads followed by *Malassezia*, *Cladosporium*, and *Phoma* (15/21, 13/21, 9/21 of samples, respectively, S2 Fig).

### Characteristics of bacterial communities between sampling locations and time points

**Fig 2:**
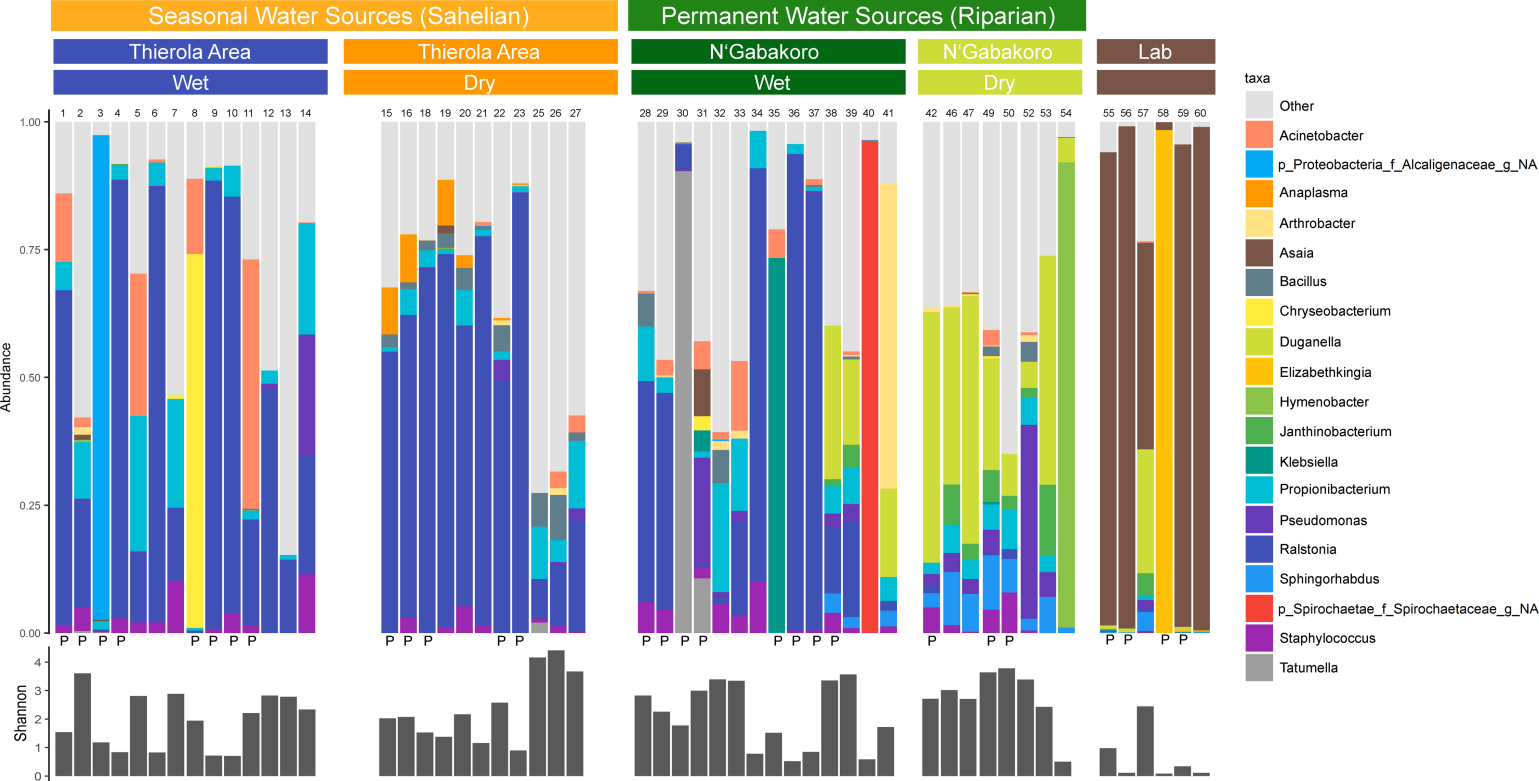
Stacked bar plot (top) and within-sample Shannon diversity (bottom) comparing the 19 most abundant agglomerated microbial genera between dry and wet seasons. All other taxa are grouped in the “other” category. Sample numbers and whether it is a pooled sample (“P”) are marked above and below bar plots, respectively. Only the top 19 genera are shown here for clarity in display and to be able to distinguish between groups, all analyses between groups were performed with all sequence variants or taxa.

The most abundant genera across field-caught mosquitoes was *Ralstonia* (25.1% of all field reads, in 42/47 samples), and the most prevalent genera was *Propionibacterium* (46/47 samples) (Fig 2). Laboratory samples were largely dominated by *Asaia* (62.9% of all laboratory reads), with this genus being the most prevalent taxa in 5/6 samples. There was no significant difference in the Shannon diversity between groups of field samples via Kruskal-Wallis chi-squared with Dunn’s multiple comparisons test, though the mean diversity of the laboratory samples was significantly lower than all field groups (S3A–S3C Fig). While overall the dry season had the highest diversity, this difference was not significantly higher than the wet season overall (dry: 2.54, wet: 2.03, *p =* 0.087, S3B Fig). The mean number of SVs per sample varied from 42.5–65.13 in the field to 20 in the laboratory, with dry season samples from both field locations being significantly different than laboratory samples (S4A Fig, *p* = 0.0117 for Thierola Area and *p =* 0.0066 for N’Gabakoro). The mean number of genera were also higher in the field than in the laboratory (27.36–42.13 in field, 14.83 in lab,S4B Fig), again with the dry seasons being significantly higher than the laboratory samples (*p* = 0.0215 for Thierola and *p =* 0.0119 for N’Gabakoro). The Pearson correlation between the overall genus abundance and frequency (across mosquitoes) was moderate (r = 0.30 and r = 0.70, P<0.001, n = 424 for native and log-transformed values), indicating that the genera more frequent across mosquitoes were more abundant (sequence reads). However, the majority of SVs and genera were sample singletons (i.e. that sequence variant or genus was present in only one sample for 91.9% of SVs and 43.7% of genera), though the majority of reads were found in non-singletons (68.0% of sequence variant reads, 98.8% of genera-level reads) (S4A and S4B Fig). This indicates there remains a high degree of heterogeneity between samples, though dominant species have some conservation between groups. Finally, there were higher mean amounts of group specific (private) SVs within the Sahel dry season (53.6 compared to 35.1, 47.5, 31.2, and 12.7 for Sahel dry, Sahel wet, riparian dry, riparian wet, and laboratory samples, respectively), however this difference was only significantly different between the laboratory samples and the two dry seasons (*p =* 0.0173 and 0.0048 for riparian dry and Sahel dry via Dunn’s test, respectively).

### Differential abundance testing of seasonality

**Fig 3:**
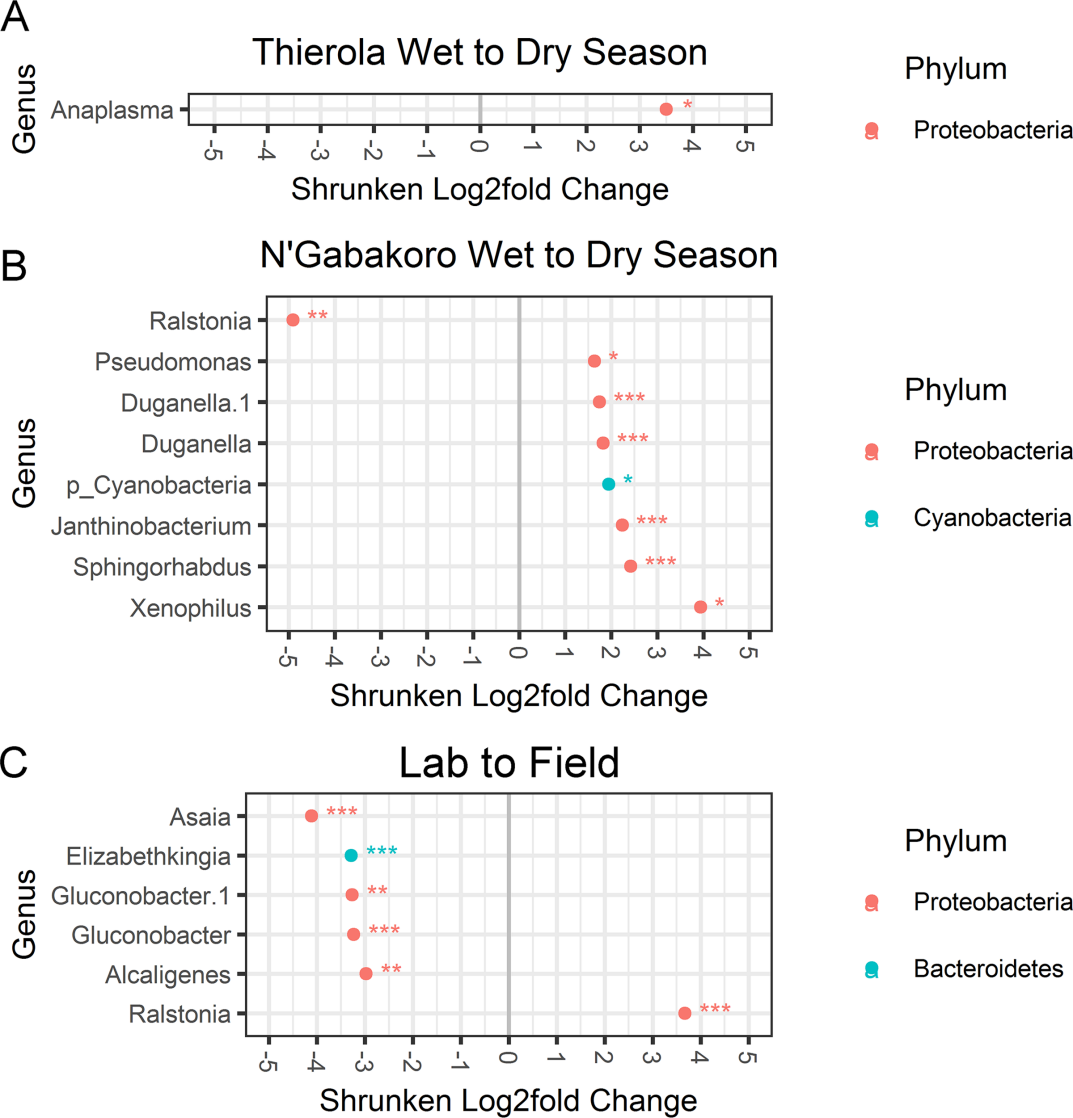
Hierarchical Multiple Testing (HMT) of differentially abundant 16s rRNA sequence variants. Log_2_ fold changes calculated via DESeq2 greater than 1.5-fold that are significant at an adjusted *p*-value < 0.05 are presented for each location. HMT is a false discovery rate adjusting methodology that arranges tested hypotheses via their phylogeny, testing sub-hypotheses only if their parent hypothesis is significant. *, **, and *** represent significance levels of *p-adj <* 0.05, 0.01 and 0.001, respectively.

Using hierarchical multiple testing on SVs to determine which SVs were differentially abundant between seasons and locations only an *Anaplasma* sequence variant was found to be differentially expressed in the dry season compared to wet in the Sahelian area (log_2_ fold-change: 3.50, *p-*adj: 0.027, Fig 3A). Eight SVs were found to be differentially expressed between seasons in the riparian location, with one more abundant in the wet season (Fig 3B, *Ralstonia*, log_2_ fold-change: -4.91, *p*-adj: 1.4e^-03^), and seven more abundant in the dry (*Pseudomonas*, two *Duganella* SVs, a *Cyanobacteria*, *Janthinobacterium*, *Sphingorhabdus*, and *Xenophilus*; log_2_ fold-change 1.63–3.94, *p-*adj: 3.19e^-02^ to 3.91e^-05^). In the laboratory versus field comparison, six SVs were found to be significant (Fig 3C, *Asaia*, *Elizabethkingia*, two *Gluconobacter* SVs, *Alcaligenes*, and *Ralstonia*, log_2_ fold-change 2.97–4.11, *p-*adj: 1.4e^-03^ to 5.61e^-15^).

### Qualitative presence/absence analysis

**Fig 4:**
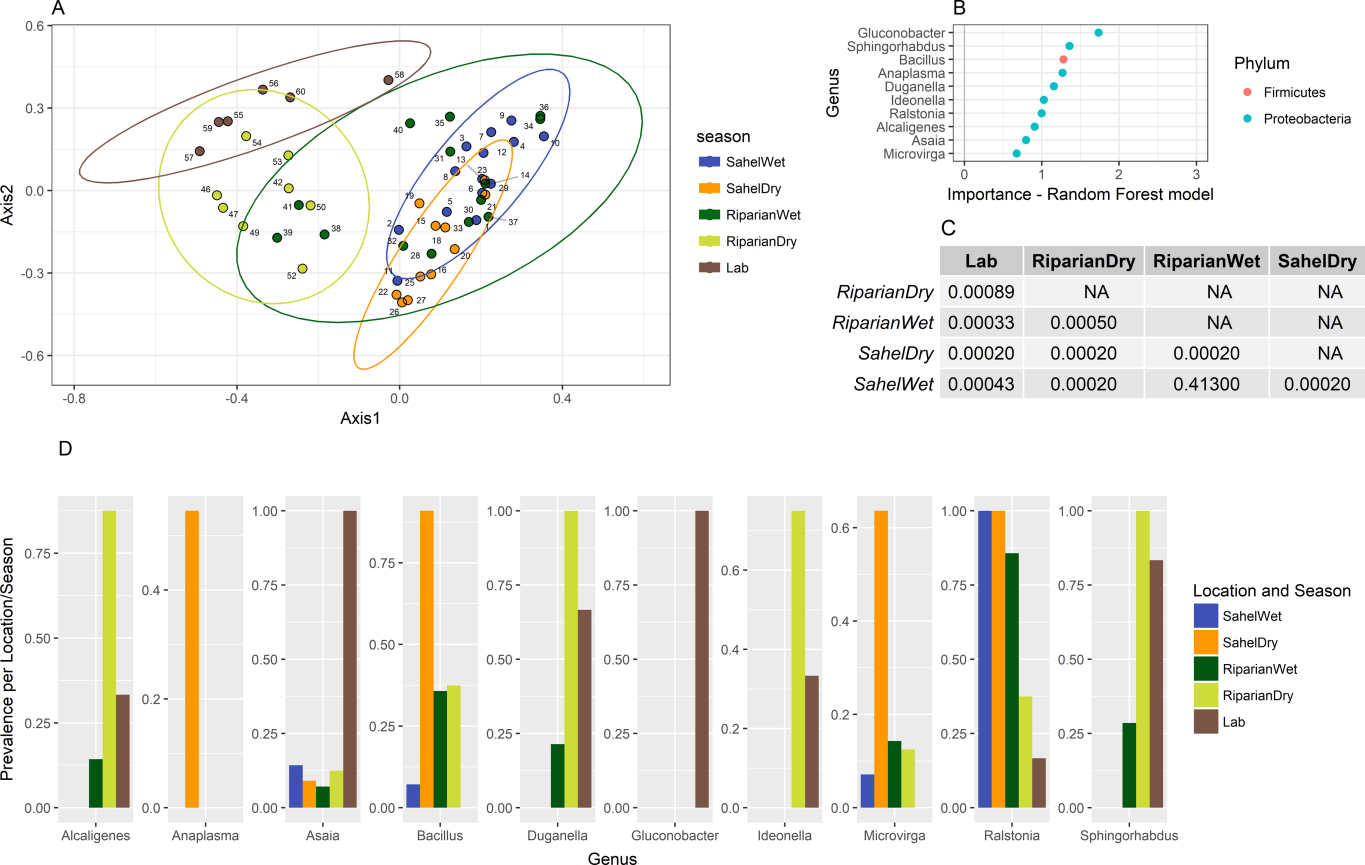
Random forest supervised learning to discriminate season and location for each sample group. Analysis was performed on all samples (pooled and individuals) for each location. The top ten variables (genera) important to the created model are shown in Panel B. Significantly different center points in the ordination via PERMANOVA are present in Panel C. Prevalence of the genera for each sample group is presented in Panel D.

We also investigated whether supervised learning approaches could discriminate dry vs. wet season samples based on the differences in presence/absence of bacteria between groups. We again found that the seasonal samples from the riparian area clustered separately more strongly than the Sahelian locations into two populations (Fig 4A), though all groups other than the wet season locations were found to have significantly different center points via PERMANOVA (Fig 4C).

To further compare the nature of the uniqueness present in the microbiome of possibly aestivating mosquitoes in the Sahelian dry season, we employed sequential testing between groups. Firstly, we tested for genera that showed significant differences between the dry and wet seasons in the Sahel. Secondly, we tested for differences between the Sahelian and riparian dry season, within the subset of genera identified earlier. Thirdly, we tested for differences between the Sahelian dry and riparian wet season within the subset that passed the first tests. In each step, we employed exact tests that accommodated sample size at the individual test level (the second and third tests were one-sided, as dictated by the direction of the difference in the first test. See methods). A total of 14 genera exhibited difference between the Sahelian dry and wet season at *p*<0.1 (with highest significance for *Bacillus* (*p*<0.00004), *Anaplasma* (*p*<0.0026), and *Microvirga* (*p*<0.007). In the subsequent tests, only *Anaplasma* which was exclusively present in the Sahelian dry season (present in 54.0% of samples vs. 0%, *p =* 0.009), *Bacillus* (90.9% vs. 7.0–38.0%, *p =* 0.020), *Intestinibacter* (45.4% vs. 0–7%, *p =* 0.022) and *Microvirga* (63.6% vs. 7.0–14.0%, *p =* 0.029) were considered putatively “characteristic” of the Sahelian dry season.

### Variation in blood feeding host preference

**Fig 5:**
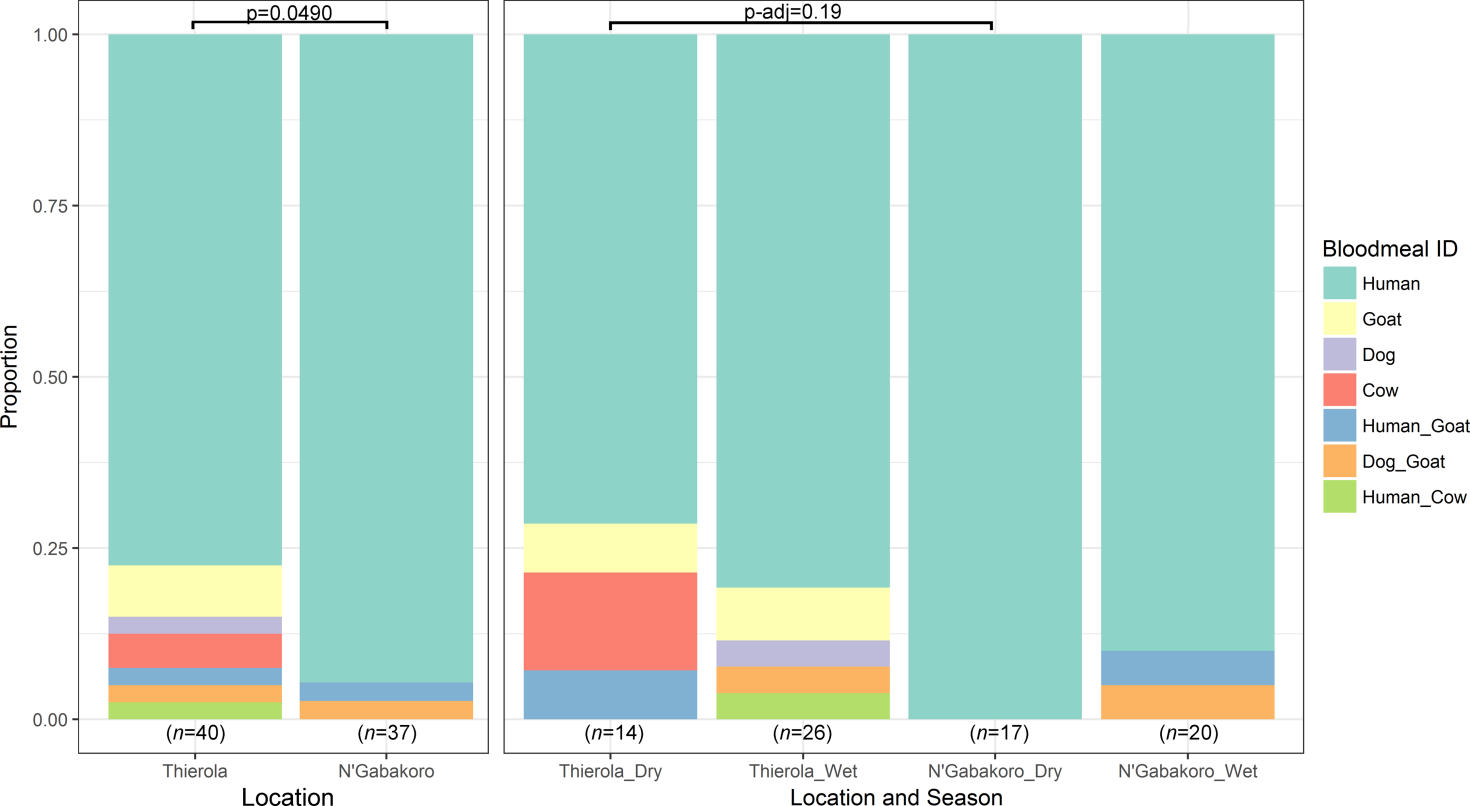
Host-choice preference determined via cytochrome B PCR. Significant difference between "human" and "other" bloodmeals for each location and season determined via a contingency table and two-tailed Fisher's exact test. *p*-values for the season and location comparison were adjusted for multiple comparisons. Bloodmeals of mixed origin are separated by an underscore, i.e. “Human_Goat” had amplification of bands corresponding to sizes of both human and goat blood.

The *Anaplasma* spp. reads found to be more prevalent during the dry season were blasted against the nr database and found to align to *Anaplasma ovis* (99–100% identity based on strain), a pathogen of goats, sheep, and wild ruminants [46]. To define the source of these reads we performed PCR to determine bloodmeal origin and the rate of anthropophily seasonally and by location. We found that Sahelian areas had a slight decrease in the degree of anthropophily overall (Fig 5 left side, 77.5% to 94.6%, comparing “Human” to all others, *p =* 0.0490 with Two-tailed Fisher’s exact test). Additionally, the Thierola area had a lower proportion of anthropophily in the dry season compared to the dry season in N’Gabakoro (71.4% human blood-feeding compared to 100%, respectively) though this difference was not significant when adjusted for multiple comparisons, *p-*adj *=* 0.19).

## Discussion

The mosquito microbiome varies over developmental stages [12,47], and has been previously shown to have distinct bacterial composition based on location and season [10,16]. In this study, we analyzed how the microbiome changes in *An*. *coluzzii* from a Sahelian area where aestivation is probable in comparison to a riparian area where aestivation is unlikely. This variation in seasonality in our sampling areas is due to a difference in latitude, causing the N’Gabakoro area to not dry out as rapidly as the more northerly areas in the Sahel, and the presence of the Niger River providing year round larval sites. Our main hypotheses were that due to the unique physiological state of Sahelian *An*. *coluzzii* mosquitoes during aestivation, the largest seasonal differences in microbial composition would be between the Sahelian dry and wet seasons, and that these characteristics would also distinguish the Sahelian dry season from either season in the riparian area [3,5,7,48]. If confirmed, aestivation-specific microbiome taxa might be used as a predictor of this state in populations where both aestivators and reproductive adults coexist.

Counter to our initial hypothesis that dry season Sahelian mosquitoes would have the most distinct microbiome composition, we found that dry season mosquitoes from the riparian area (N’Gabakoro) were, overall, the most different among our mosquito groups (described in detail below). The Sahelian dry season did exhibit some unique characteristics including high microbial diversity with highest number of private SVs, and had slight differences in overall microbial composition (Fig 4), though these differences were not as pronounced as those between seasons in permanent water locations. Additionally, several bacterial genera were significantly elevated in the Sahelian dry season including *Anaplasma* (exclusively present in the Sahelian dry season), *Bacillus* (in 90.9% of Sahelian dry samples vs. 7–37.5% in others), *Microvirga* (63.6% vs. 7–14%), and *Intestinibacter* (45.4% vs. 0–7%). We also note that three of these genera (*Anaplasma*, *Bacillus*, and *Microvirga*) were also found to be important to the random forest classification model (Fig 4B and 4D). Only *Anaplasma* was found to be significantly different based on read count abundance (log_2_ fold-change: 3.50, *p-*adj: 0.027, Fig 3). The most likely route of acquisition of *Bacillus*, *Microvirga*, and *Intestinibacter* would be through the larval environment or possibly plant feeding due to their soil/water association (S1 Table). As no private genera were present in all Sahelian dry season mosquitoes, if bacterial biomarkers of aestivation exist, it may indicate not all mosquitoes in this period are in this state or that alternative but rare species can play the same role. Further studies are necessary to test whether these putative differences are indeed “characteristic” of the Sahelian dry season and has relevance to aestivation. Ideally, these studies will characterize the reproducibility of the interactions between years.

The riparian dry season sample set was the most unique of the field samples and was characterized by a reduction of *Ralstonia* reads, and an increase in *Duganella*, *Janthinobacterium*, and *Sphingomonas* reads as determined by hierarchical multiple testing and ordination (Figs 3 and 4B). The most abundant genera in wild-caught samples was *Ralstonia* (present in 40/47, most abundant taxa in 24/47), and had little representation in laboratory mosquitoes (1/6 samples with 10 reads). This genus has been seen previously in wild-caught *Anopheles* from Cameroon [13] and *Aedes aegypti* [49], and is believed to be largely soil and water associated [50,51]. Additionally, we found that the majority of 16S SVs were unique to an individual sample (91.9%, S5 Fig), indicating that there is a high degree of heterogeneity between mosquitoes. However these SVs were relatively low in abundance, only accounting for 32.0% of reads. These SVs also demonstrate the additional sub-species level heterogeneity that the more commonly used operational taxonomic units (OTUs) may miss. A caveat to this finding is that due to the low read counts of the sequencing technology used, the high percentage of uniqueness of SVs may be linked more to this limited coverage rather than biology.

As it has been previously reported in *Aedes aegypti* that most of the adult gut flora is acquired transstadially from larval stages to adults [47], and the “core microbiome” does not change significantly in laboratory adults released into the wild and recaptured [52]. This may help explain why there were limited seasonal differences in mosquitoes in the northern Sahel. Due to the prolonged dry season, there are no known larval sites available during this period [5]. Thus, the mosquitoes present would have been larvae in the same rainfall-linked transient water sources that the wet season mosquitoes developed in, and any changes present in the microbiome would be due to incorporation of new flora from adult environmental conditions, or blood/sugar sources. This homogeneity in larval environments is contrasted in the available larval habitat near N’Gabakoro that changes broadly throughout the year, from fresh rain puddles to ground and river water in rock and other pools near the Niger River that develop as flooded areas recede [53,54], or from standing water present in the more urban area of Bamako nearby (though these pools are likely unsuitable for *Anopheles* spp. growth). This explanation needs to be tested beyond this work, however it could explain why the dry season Sahelian samples had the most pronounced difference in genera likely acquired through blood feeding, and the riparian areas had the largest differences in bacteria that are predominantly soil and water associated (S1 Table). This may also explain why the previous analysis of seasonality in a wetter climate of Ghana showed limited seasonal differences in *Anopheles coluzzii* [16]. Another difference between the studies is related to the Ghanaian study use of day 1 post emergence mosquitoes that were collected as larvae in differing seasonal water sources and were non-bloodfed [16]. This may then limit effects of the adult environment upon the microbiome. Finally, an additional possibility is that mosquitoes collected during the dry season peak in April are failed aestivators who are exiting from this state prior to the wet season or migrants from distant larval habitat [7]. Due to the high, synchronous density and aggregation of emergence however, these are unlikely to be the main source [55]. Future studies should compare water and larval samples from collection locations to further refine what is the main route of acquisition of these genera, and whether distinct seasonal patterns are consistent between years.

Towards determining the possible route of acquisition of the only Sahelian dry season specific bacteria found, we looked further into the nature of *Anaplasma ovis* and its known hosts. *A*. *ovis* 16S sequence has been found previously in *Anopheles gambiae* and *An*. *funestus* collected in western Kenya [56], though it remains unknown if this finding has any relevance to the disease’s transmission or is simply related to it being an intracellular pathogen of the blood that could be inadvertently picked up during feeding. Towards this, the analysis of host-choice PCR with cytochrome B allowed us to refine the biting characteristics of each population across seasons. We found that the relaxation of strict anthropophily in the seasonal areas (Fig 5) which may follow what has been reported previously in areas of low host-availability [57]. Due to the climactic severity during the dry season in the Sahel, the acquisition of blood from the nearest source is likely less taxing than feeding on the preferred host. Though as there is not an increase in zoophily in the N’Gabakoro samples in March-April in the dry season (when conditions are similar between locations) and there is some zoophily in the wet season, there may be an innate degree of zoophily in all *An*. *coluzzii* even in favorable conditions. This could also better explain the presence of *Anaplasma ovis* in these samples as being incidentally acquired from a more catholic feeding behavior in mosquitoes thought to be primarily anthropophilic [58]. Furthermore, *Anaplasma phagocytophilum* has been shown to modulate *Ixodes* tick microbiota, though the effects of *Anaplasma* on mosquito microbiota has not been studied to our knowledge [59]. This pathogen is also in the same order as the *Wolbachia* genus bacteria that have achieved considerable attention as an *Aedine* symbiont and possible vectorial-competence modifying species, but again its effects upon the mosquito vector are currently unknown [60–62].

A caveat to our microbiome composition data is that the sampling and next-generation sequencing in this study was performed prior to the widespread description of “kitome” DNA present from DNA extraction kits, molecular water, and cross-over contamination on microbiome analysis [63]. Due to this, we do not have control samples from each of these items, and due to the time since processing, these kits are no longer available to be sequenced. The issues of contamination have been reported to be more severe with samples of low-bacterial abundance [63]. Following this association, we found that there were no significant negative correlations between read count and presence of known kitome genera as would be expected from this sort of contamination (two-tailed Pearson’s r, S2 Table). This lack of correlation should not imply there is no contamination possibility in our samples, but is the best assessment we can provide retrospectively that presence of these genera is not linked to low-biomass in our samples. Additionally, no reads were broadly found in all samples, and all samples were extracted and PCR amplified at the same time using the same kits. Future studies should include these negative controls, and assessment of aquatic bacterial species from the larval sites to limit these possible confounders. Additionally, use of newer versions of next-generation sequencing approaches could also help to improve sequencing coverage, allowing for more efficient filtering of these low prevalence reads.

## Conclusions

This study of the seasonal variation in the microbiome of *An*. *coluzzii* from Sahelian and riparian habitats was designed to explore if there are major differences that could link the state of aestivation to a particular composition of the microbiome. While notably, the diversity of the microbiome of individual mosquitoes is very large with >90% sequence variant singletons and this diversity increases in the dry season, when considering the main differences in microbiome composition we do not find the Sahelian samples to follow our hypothesis and instead suggests that the riparian dry season sample is the most distinct. We interpret these results to suggest that the most abundant and frequent members of the microbiome are not well linked to aestivation or that the specimens used here do not represent the microbiome of the aestivating mosquitoes [3,7,48]. Finally, it is also possible that the main changes in the mosquito microbiome depend on its larval environment and not on this physiological state. Accordingly, the greater seasonal difference in the microbiome of the riparian habitat reflects the change from typical–“ephemeral puddles” prevalent in the wet season (in both Sahelian and riparian habitats) to the more permanent larval sites that form in the bank of the river as it recedes in the riparian area. This inversion of our original hypothesis with respect to the magnitude of the seasonal difference between habitats, requires additional studies to discern between these new explanations.

## Supporting information

S1 Fig Schematic of prevalence testing for bacterial genera between species. (TIF)

S2 Fig Seasonal fungal biome of mosquitoes determined via ITS region sequencing. “P” denotes samples that are pooled from 3 individuals, all others are from individual mosquitoes.
(TIF)

S3 Fig Comparison of Shannon diversity between samples groups. Changes between season/location (A), season (B), and field vs. lab (C) are shown. Significance determined via Kruskal-Wallis chi-square with Dunn’s multiple comparison adjustment where applicable. *, **, and *** represent significance levels of *p-adj <* 0.05, 0.01 and 0.001, respectively.
(TIF)

S4 Fig Comparison of mean number of sequence variants (A) and genera (B) for each season/location. Significance determined via Kruskal-Wallis chi-square with Dunn’s multiple comparison adjustment where applicable. *, **, and *** represent significance levels of *p-adj <* 0.05, 0.01 and 0.001, respectively.
(TIFF)

S5 Fig **Histogram showing the number of samples each genus (A) or sequence variant (B) were present in.** The y-axis shows how many genera or sequence variants fall into that group (i.e. how many were in one sample, two samples, etc.).
(TIFF)

S1 Table Supplementary information on the 19 most abundant genera in the study. Presence in arthropods is from Minard *et al*. unless otherwise noted. Non-insect environment and presence in arthropods is not comprehensive.
(DOCX)

S2 Table No correlation of known “kitome” genera with read count in our study. Kitome genera are based on summary table from Salter *et al*.
(DOCX)

S3 Table Meta-data and read count of samples. (CSV)
