# Supplementary figures and images for: Investigation of the seasonal microbiome of Anopheles coluzzii mosquitoes in Mali
Source: PLoS One. 2018 Mar 29;13(3):e0194899. doi: 10.1371/journal.pone.0194899 (PMC5875798; doi:10.1371/journal.pone.0194899)

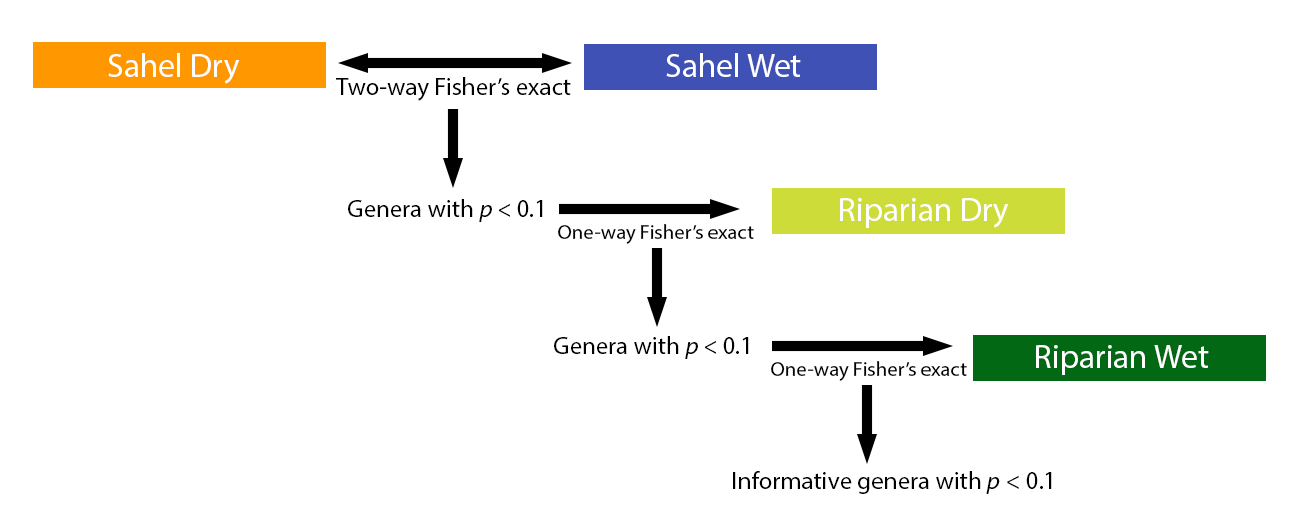

Supplement: S1 Fig — (TIF) [file pone.0194899.s001.tif]

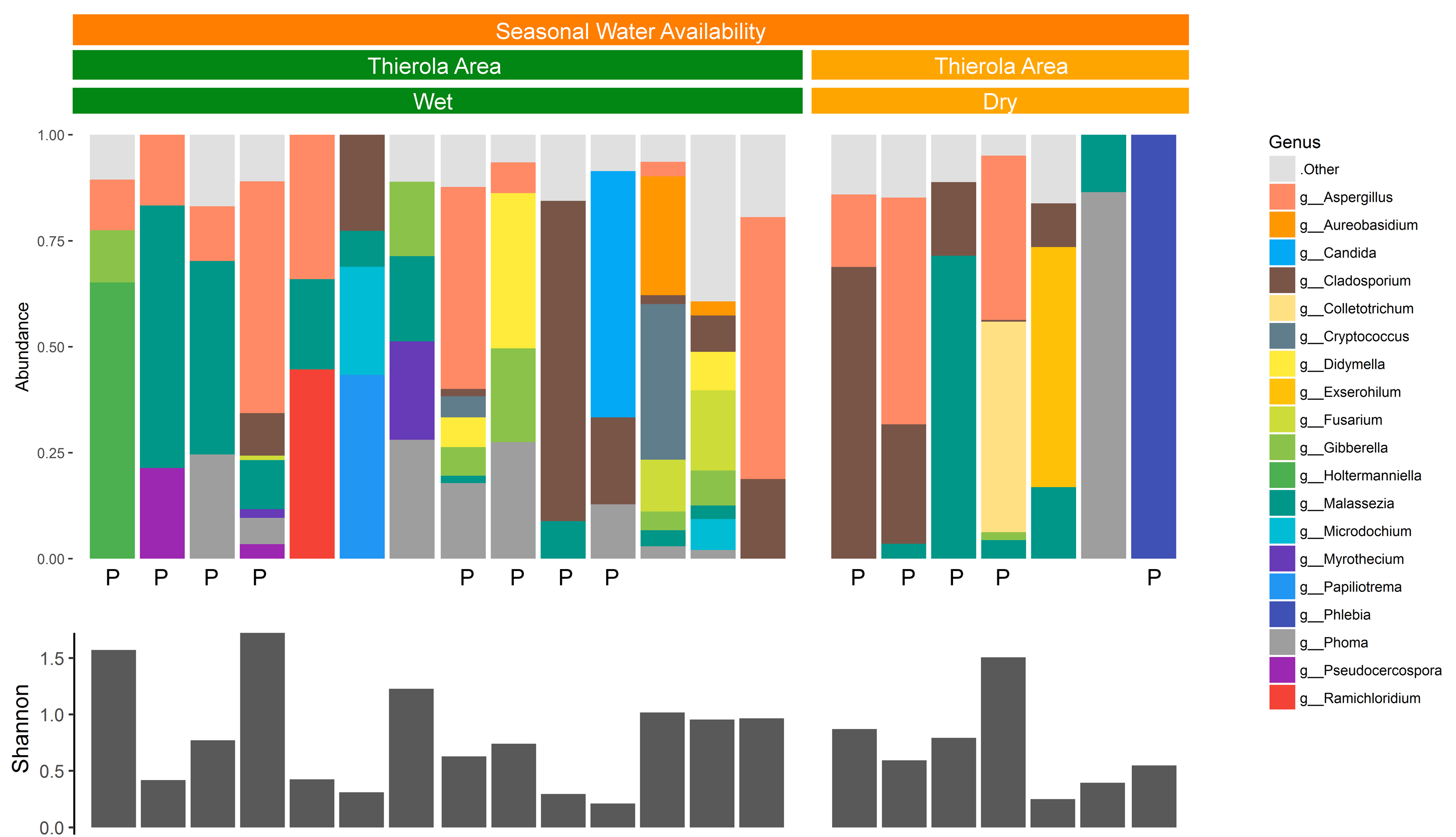

Supplement: S2 Fig — “P” denotes samples that are pooled from 3 individuals, all others are from individual mosquitoes. (TIF) [file pone.0194899.s002.tif]

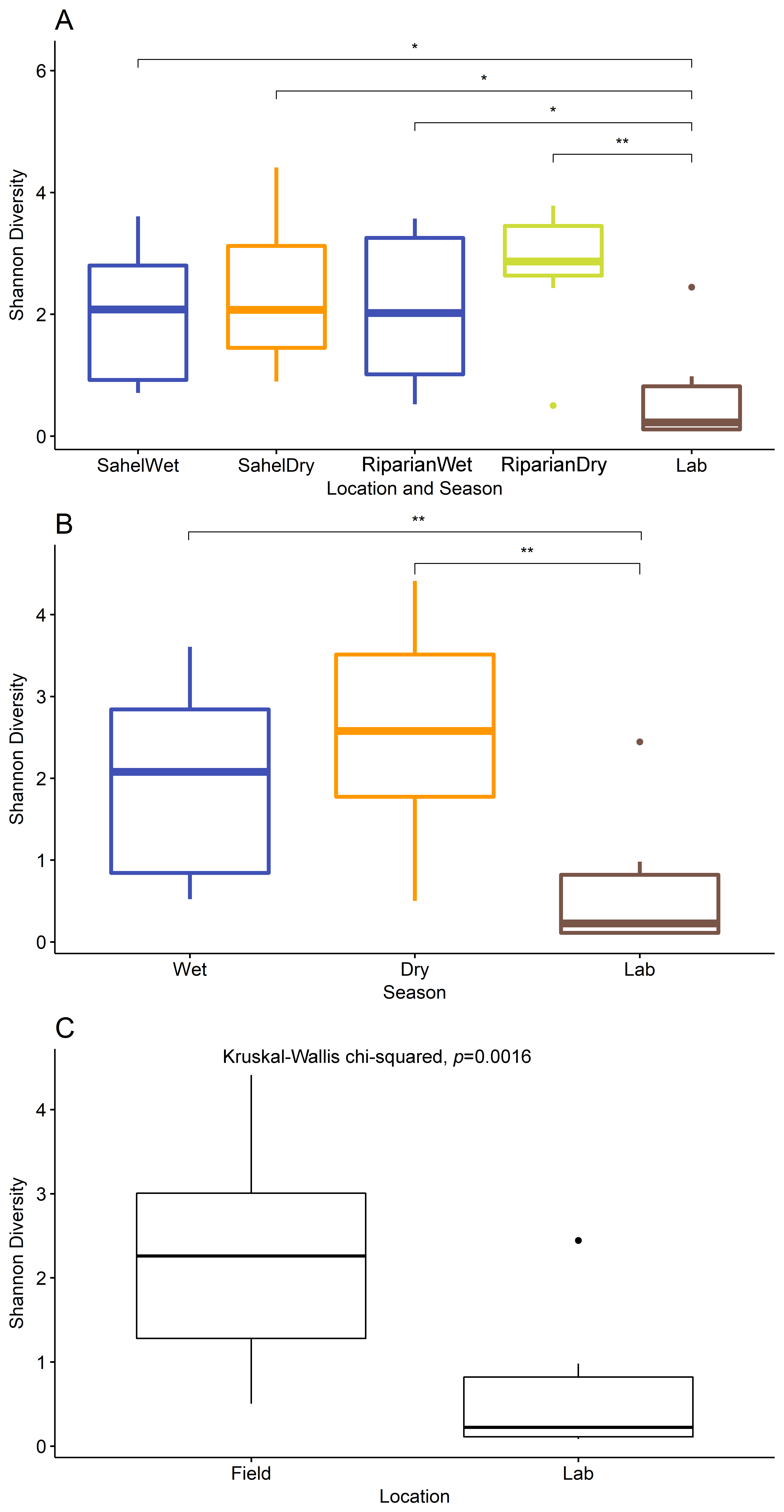

Supplement: S3 Fig — Changes between season/location (A), season (B), and field vs. lab (C) are shown. Significance determined via Kruskal-Wallis chi-square with Dunn’s multiple comparison adjustment where applicable. *, **, and *** represent significance levels of p-adj < 0.05, 0.01 and 0.001, respectively. (TIF) [file pone.0194899.s003.tif]

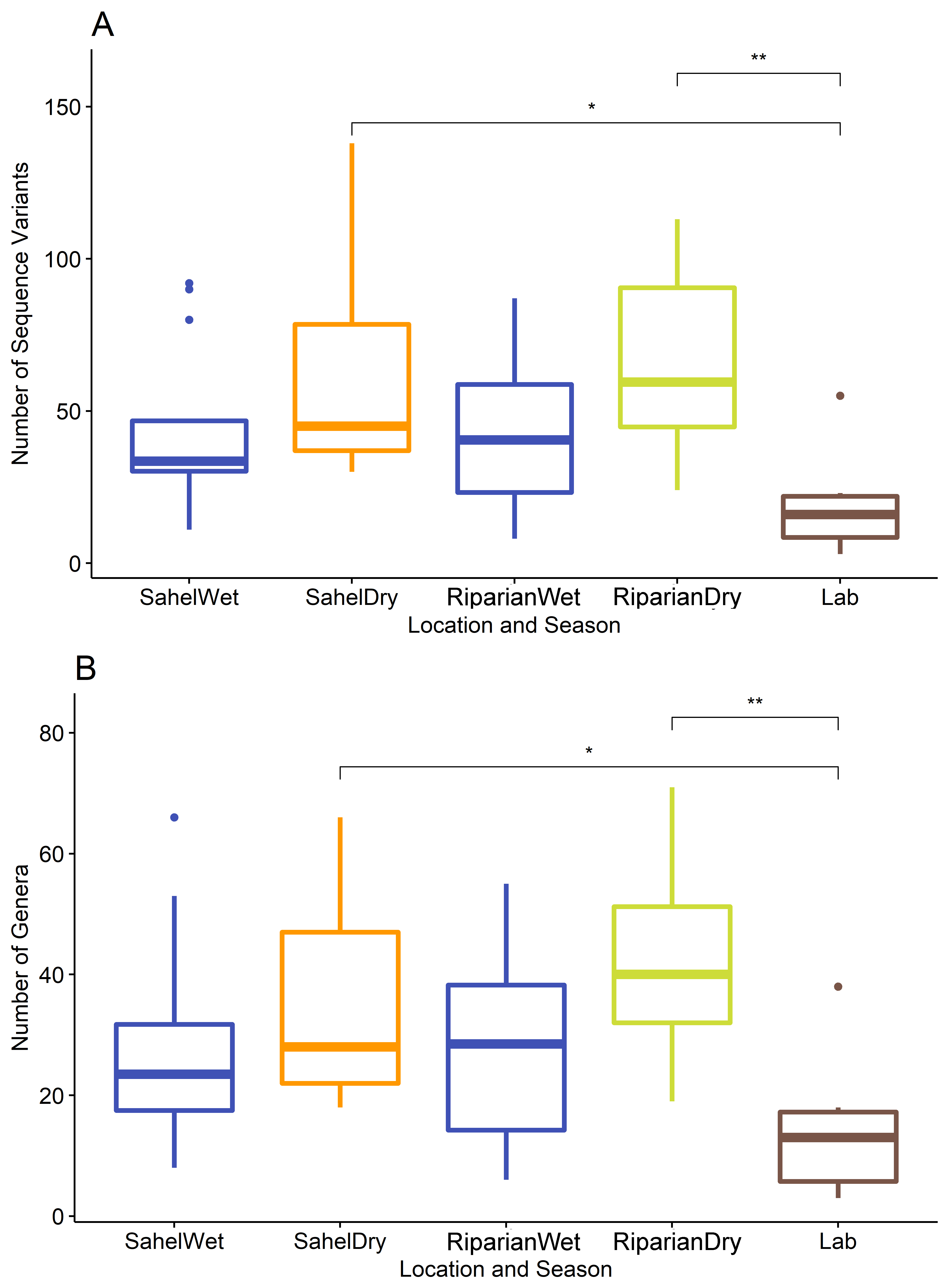

Supplement: S4 Fig — Significance determined via Kruskal-Wallis chi-square with Dunn’s multiple comparison adjustment where applicable. *, **, and *** represent significance levels of p-adj < 0.05, 0.01 and 0.001, respectively. (TIFF) [file pone.0194899.s004.tiff]

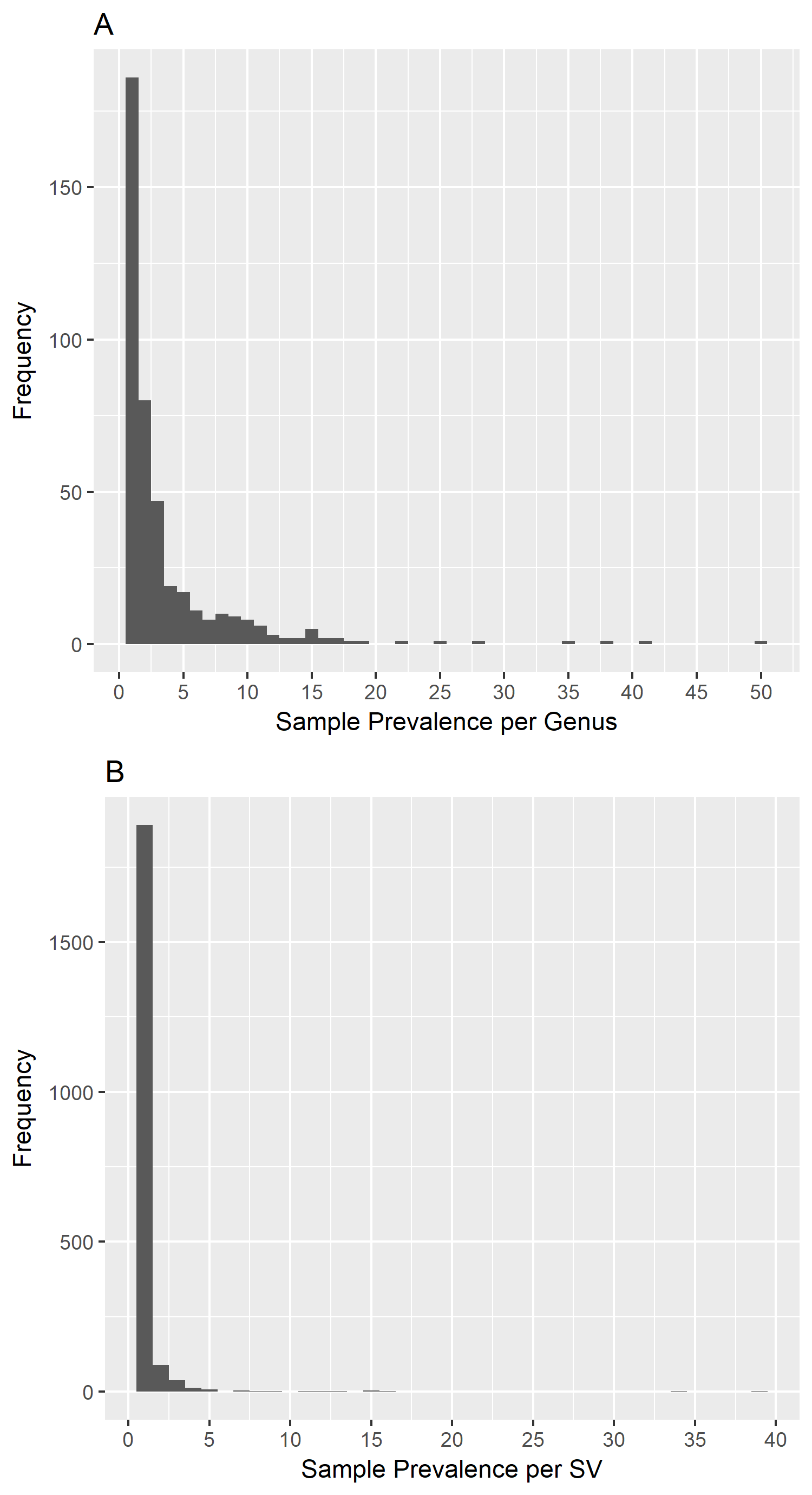

Supplement: S5 Fig — Histogram showing the number of samples each genus (A) or sequence variant (B) were present in. The y-axis shows how many genera or sequence variants fall into that group (i.e. how many were in one sample, two samples, etc.). (TIFF) [file pone.0194899.s005.tiff]
