# Supplementary material for: Investigation of the seasonal microbiome of Anopheles coluzzii mosquitoes in Mali
Source: PLoS One. 2018 Mar 29;13(3):e0194899. doi: 10.1371/journal.pone.0194899 (PMC5875798; doi:10.1371/journal.pone.0194899)
Supplement: S1 Table — Presence in arthropods is from Minard et al. unless otherwise noted. Non-insect environment and presence in arthropods is not comprehensive. (DOCX) [file pone.0194899.s006.docx]

**Table S1: Supplementary information on the 19 most abundant genera in the study.** Presence in arthropods is from Minard *et al.* unless otherwise noted. Non-insect environment and presence in arthropods is not comprehensive.

| Family | Genera | Possible species (nr BLAST) | Non-insect Environment | Type | Seen in arthropods?^1^ |
| --- | --- | --- | --- | --- | --- |
|  | *Acinetobacter* | *soli,* c*alcoaceticus*,  or *baumannii (100%)* | soil ^2^; nosocomial^3^;  Plants/nectar^4^ | gram-negative aerobic | *Aedes, Anopheles, Culex, Mansonia, Psorophora* |
| *Alcaligenaceae* |  | *faecalis (95%)* | feces, water, and soil^5^ | gram-negative aerobic | *An. stephensi* |
|  | *Anaplasma* | *ovis (100%)* | obligate intracellular blood pathogen -- ruminants^6^ | gram-negative aerobic | *An. gambiae*^7^*, Ticks*^6^ |
|  | *Arthrobacter* | *agilis (99%)* | Soil, water, human skin^8^ | gram-positive aerobic | *Aedes, Culex* |
|  | *Asaia* | *siamensis, bogorensis, or krungthepensis (100%)* | tropical plants^9^ | gram-negative aerobic | *Aedes, Anopheles,  Mansonia, Culex* |
|  | *Bacillus* | *kyonggiensis (100%)* | ubiquitous, soil^10^ | gram-positive aerobic | *Aedes, Anopheles,  Mansonia, Culex* |
|  | *Chryseobacterium* | *indologenes (99%)* | soil, plants, foodstuffs, water^11^ | gram-negative anaerobic | *Aedes, Anopheles,  Mansonia, Culex* |
|  | *Duganella* | *N/A* | soil and water^12^ | gram-negative aerobic | *Asobara spp.*wasps^13^,  *Agapeta* moths^14^ |
|  | *Elizabethkingia* | *meningoseptica (100%)* | ubiquitous, water, soil, nosocomial^15^ | gram-negative aerobic | *Anopheles, Aedes* |
|  | *Hymenobacter* | *N/A* | soil^16^, airborne^17^,  water associated with *Anopheles* larvae^18^ | gram-negative aerobic | *Amblyomma* ticks^19^ |
|  | *Janthinobacterium* | *lividum (99%)* | soil^20^ | gram-negative aerobic | *Aedes* |
|  | *Klebsiella* | *pneumoniae* or  *variicola (100%)* | soil, mouth, skin, intestines, plants^21^ | gram-negative anaerobic | *Aedes, Anopheles, Culex* |
|  | *Propionibacterium* | *acnes (100%)* | skin^22^ | gram-positive anaerobic | *Aedes, Anopheles, Mansonia* |
|  | *Pseudomonas* | *5+ species (100%)* | soil, water^23^ | gram-negative aerobic | *Aedes, Anopheles, Culex,  Mansonia, Psorophora* |
|  | *Ralstonia* | *insidiosa* or  *picketii (100%)* | soil, water^24^ | gram-negative aerobic | *Aedes, Anopheles,* Habitat |
|  | *Sphingorhabdus* | *uncultured (100%)* | soil | gram-negative aerobic | N/A |
| *Spirochaetaceae* |  | *Spironema culicis (96%)* | insect symbiont | gram-negative aerobic | *Culex, Simuliidae* black flies^25,26^ |
|  | *Staphylococcus* | *uncultured (100%)* | ubiquitous | gram-positive facultative anaerobic | *Aedes, Anopheles, Culex, Mansonia* |
|  | *Tatumella* | *uncultured* or *g_Rosenbergiella (99%)* | plant/nectar^27^ | gram-negative anaerobic | N/A |

1. Minard, G. *et al.* Pyrosequencing 16S rRNA genes of bacteria associated with wild tiger mosquito Aedes albopictus: a pilot study. *Front. Cell. Infect. Microbiol.* **4,** 59 (2014).

2. Kim, D. *et al.* Acinetobacter soli sp. nov., isolated from forest soil. *J. Microbiol.* **46,** 396–401 (2008).

3. Bergogne-Berezin, E. The Increasing Role of Acinetobacter Species As Nosocomial Pathogens. *Curr Infect Dis Rep* **3,** 440–444 (2001).

4. Álvarez-Pérez, S., Lievens, B., Jacquemyn, H. & Herrera, C. M. Acinetobacter nectaris sp. nov. and Acinetobacter boissieri sp. nov., isolated from floral nectar of wild Mediterranean insect-pollinated plants. *Int. J. Syst. Evol. Microbiol.* **63,** 1532–1539 (2013).

5. Rehfuss, M. & Urban, J. Alcaligenes faecalis subsp. phenolicus subsp. nov. a phenol-degrading, denitrifying bacterium isolated from a graywater bioprocessor. *Syst. Appl. Microbiol.* **28,** 421–429 (2005).

6. de la Fuente, J. *et al.* Sequence analysis of the msp4 gene of Anaplasma ovis strains. *Vet. Microbiol.* **119,** 375–381 (2007).

7. Lindh, J. M. *et al.* 16S rRNA Gene-Based Identification of Midgut Bacteria from Field-Caught *Anopheles gambiae* Sensu Lato and *A. funestus* Mosquitoes Reveals New Species Related to Known Insect Symbionts. *Appl. Environ. Microbiol.* **71,** 7217–7223 (2005).

8. Koch, C., Schumann, P. & Stackebrandt, E. Reclassification of Micrococcus agilis (Ali-Cohen 1889) to the Genus *Arthrobacter* as *Arthrobacter agilis* comb. nov. and Emendation of the Genus *Arthrobacter*. *Int. J. Syst. Bacteriol.* **45,** 837–839 (1995).

9. Favia, G. *et al.* Bacteria of the Genus Asaia: A Potential Paratransgenic Weapon Against Malaria. in *Transgenesis and the Management of Vector-Borne Disease* 49–59 (2008).

10. Dong, K. & Lee, S. Bacillus kyonggiensis sp. nov., isolated from soil of a lettuce field. *J. Microbiol.* **49,** 776–781 (2011).

11. Montero-Calasanz, M. del C. *et al.* Chryseobacterium hispalense sp. nov., a plantgrowth- promoting bacterium isolated from a rainwater pond in an olive plant nursery, and emended descriptions of Chryseobacterium defluvii, Chryseobacterium indologenes, Chryseobacterium wanjuense and Chryseob. *Int. J. Syst. Evol. Microbiol.* **63,** 4386–4395 (2013).

12. Li, W. J. *et al.* Duganella violaceinigra sp. nov., a novel mesophilic bacterium isolated from forest soil. *Int. J. Syst. Evol. Microbiol.* **54,** 1811–1814 (2004).

13. Zouache, K., Voronin, D., Tran-Van, V. & Mavingui, P. Composition of bacterial communities associated with natural and laboratory populations of Asobara tabida infected with Wolbachia. *Appl. Environ. Microbiol.* **75,** 3755–3764 (2009).

14. Frederick, B. & Caesar, A. Analysis of bacterial communities associated with insect biological control agents using molecular techniques. *Proc. X Int. Symp. Biol. Control Weeds* **267,** 261–267 (2000).

15. Hsu, M. S. *et al.* Clinical features, antimicrobial susceptibilities, and outcomes of Elizabethkingia meningoseptica (Chryseobacterium meningosepticum) bacteremia at a medical center in Taiwan, 1999-2006. *Eur. J. Clin. Microbiol. Infect. Dis.* **30,** 1271–1278 (2011).

16. Kim, K. H., Im, W. T. & Lee, S. T. Hymenobacter soli sp. nov., isolated from grass soil. *Int. J. Syst. Evol. Microbiol.* **58,** 941–945 (2008).

17. Yooseph, S. *et al.* A metagenomic framework for the study of airborne microbial communities. *PLoS One* **8,** (2013).

18. Gimonneau, G. *et al.* Composition of Anopheles coluzzii and Anopheles gambiae microbiota from larval to adult stages. *Infect. Genet. Evol.* **28,** 715–724 (2014).

19. Trout Fryxell, R. T. & DeBruyn, J. M. The Microbiome of Ehrlichia-Infected and Uninfected Lone Star Ticks (Amblyomma americanum). *PLoS One* **11,** e0146651 (2016).

20. Shivaji, S. *et al.* Identification of Janthinobacterium-Lividum From the Soils of the Islands of Scotia Ridge South Atlantic and From Antarctic Peninsula. *Polar Biol.* **11,** 267–272 (1991).

21. Brown, C. & Seidler, R. J. Potential pathogens in the environment: Klebsiella pneumoniae, a taxonomic and ecological enigma. *Appl. Microbiol.* **25,** 900–904 (1973).

22. Brüggemann, H. *et al.* The Complete Genome Sequence of Propionibacterium Acnes, a Commensal of Human Skin. *Science (80-. ).* **305,** 671–673 (2004).

23. Nelson, K. E. *et al.* Complete genome sequence and comparative analysis of the metabolically versatile Pseudomonas putida KT2440. *Environ. Microbiol.* **4,** 799–808 (2002).

24. Coenye, T., Goris, J., De Vos, P., Vandamme, P. & LiPuma, J. J. Classification of Ralstonia pickettii-like isolates from the environment and clinical samples as Ralstonia insidiosa sp. nov. *Int. J. Syst. Evol. Microbiol.* **53,** 1075–1080 (2003).

25. Čechová, L., Durnová, E., Šikutová, S., Halouzka, J. & Němec, M. Characterization of spirochetal isolates from arthropods collected in South Moravia, Czech Republic, using fatty acid methyl esters analysis. *J. Chromatogr. B Anal. Technol. Biomed. Life Sci.* **808,** 249–254 (2004).

26. Šikutov, S., Halouzka, J., Mendel, J., Knoz, J. & Rudolf, I. Novel spirochetes isolated from mosquitoes and black flies in the Czech Republic. *J. Vector Ecol.* **35,** 50–55 (2010).

27. Halpern, M., Fridman, S., Atamna-Ismaeel, N. & Izhaki, I. Rosenbergiella nectarea gen. nov., sp. nov., in the family Enterobacteriaceae, isolated from floral nectar. *Int. J. Syst. Evol. Microbiol.* **63,** 4259–4265 (2013).
