# Supplementary material for: Investigation of the seasonal microbiome of Anopheles coluzzii mosquitoes in Mali
Source: PLoS One. 2018 Mar 29;13(3):e0194899. doi: 10.1371/journal.pone.0194899 (PMC5875798; doi:10.1371/journal.pone.0194899)
Supplement: S2 Table — Kitome genera are based on summary table from Salter et al. (DOCX) [file pone.0194899.s007.docx]

**Table S2: No correlation of known “kitome” genera with read count in our study.** Kitome genera are based on summary table from Salter *et al.* [1]

| **Genera** | **Pearson's r** | **R^2^ value** | **P-value (two-tailed)** |
| --- | --- | --- | --- |
| Acinetobacter | 0.1026 | 0.0105 | 0.4645 |
| Arthrobacter | -0.09141 | 0.0084 | 0.5151 |
| Bacillus | -0.1723 | 0.0297 | 0.2174 |
| Duganella | 0.02112 | 0.0004 | 0.8807 |
| Propionibacterium | -0.01913 | 0.0366 | 0.1501 |
| Pseudomonas | -0.05678 | 0.0032 | 0.6863 |
| Ralstonia | -0.2166 | 0.0469 | 0.1192 |

1. Salter SJ, Cox MJ, Turek EM, Calus ST, Cookson WO, Moffatt MF, et al. Reagent and laboratory contamination can critically impact sequence-based microbiome analyses. BMC Biol. 2014;12: 87. doi:10.1186/s12915-014-0087-z
